# Sensoring the Neck: Classifying Movements and Actions with a Neck-Mounted Wearable Device †

**DOI:** 10.3390/s22124313

**Published:** 2022-06-07

**Authors:** Jonathan Lacanlale, Paruyr Isayan, Katya Mkrtchyan, Ani Nahapetian

**Affiliations:** Computer Science Department, California State University, Northridge (CSUN), Northridge, CA 91330, USA; jonathan.lacanlale.608@my.csun.edu (J.L.); paruyr.isayan.536@my.csun.edu (P.I.); katya.mkrtchyan@csun.edu (K.M.)

**Keywords:** wearable computing, interaction design, neck-mounted interface, flex sensor, machine learning (ML)

## Abstract

Sensor technology that captures information from a user’s neck region can enable a range of new possibilities, including less intrusive mobile software interfaces. In this work, we investigate the feasibility of using a single inexpensive flex sensor mounted at the neck to capture information about head gestures, about mouth movements, and about the presence of audible speech. Different sensor sizes and various sensor positions on the neck are experimentally evaluated. With data collected from experiments performed on the finalized prototype, a classification accuracy of 91% in differentiating common head gestures, a classification accuracy of 63% in differentiating mouth movements, and a classification accuracy of 83% in speech detection are achieved.

## 1. Introduction

The ever-increasing prevalence of mobile phones, wearable devices, and smart speakers has spurred intense exploration into user interfaces. These new user interfaces need to address the challenges posed by the ubiquitous interaction paradigm, while having available the possibilities that these varied smart technologies provide.

Arenas for exploration of mobile user interfaces include improving gesture-based interfaces to enable interaction in limit mobility settings or by decreasing the social disruption that is caused by repeated disruptive interactions. Interfaces have been developed that use the movement of the hands, arms, eyes, and feet.

Touch gesture controls still dominate mobile system interfaces because of the ubiquity of touch screens [1]. However, the dominant tap, scroll, and pinch gestures have been linked to repetitive strain injuries on smart phones [2,3]. In addition, they have their limitations on wearable devices because of the limited screen size and, in turn, the available interface surface. The gestures on smartwatch screens need to be done with greater precision and with more constriction of the hand muscles, since the smartwatch screens are significantly smaller than the smartphone screens.

Voice user interfaces (VUIs) that are used for smart speakers have been another arena for improvement, with voiceless speech being explored for situations where there is background noise and for microinteractions.

In this work, we examine the benefits that sensoring the neck can provide within the breadth of mobile user interfaces. We explore and develop a new user interface for mobile systems, independent of limb motions. For example, in place of a scroll down, the head can be tilted forward. In place of a tap, the head can be turned to one side, all with only an inexpensive sensor affixed to the neck or shirt collar.

We sensor the neck with an inexpensive and nonintrusive flex sensor and show the range of interfaces that are possible with the incorporation of this simple wearable technology into our lives. Our efforts provide a proof of concept that common actions, such as head tilts, mouth movements, and even speech, can be classified through the interpretation of the bend angle received from the neck. We explore the size of the flex sensor and the positioning of the sensor on the neck and use our classification results to tailor the prototype.

Applications for neck interfaces include use in assistive devices where limb motion is limited, in gaming and augmented reality systems for more immersive experiences, and in wearable and vehicular systems where hand and/or voice use is restricted or inconvenient. Neck interactions expand a user’s bandwidth for information transference, in conjunction with or in place of the typically saturated visual and the audial channels.

A neck-mounted prototype was designed and developed, as detailed in Section 3. The system design considered comfort and the range of motion in the neck and upper body. The form factor and the positioning of the system was finalized to enable the embedding in clothing, such as in a shirt collar. A range of sensor types, sizes, and positions were considered and evaluated.

The prototype’s head gesture and position classification accuracy was evaluated for five different classes of common head tilt positions. These experimental evaluations are detailed in Section 4. Head tilt classification is important because it enables user interface input with simple and subtle head gestures. 

The encouraging results from the head gesture classification motivated us to explore more possibilities, including using the prototype for mouth movement and speech classification. The experimental evaluations of mouth movements and speech classification are detailed in Section 5. By also incorporating speech and/or mouth movement detection, head gestures for software interactions can be differentiated from head gestures that arise during regular conversation.

The main contributions of this work are (1) the development of a neck-mounted prototype, with an evaluation of sensor types, sizes, and positions; (2) the evaluation of the prototype’s head-position classification accuracy; (3) mouth movement detection; and (4) speech detection and classification.

## 2. Related Work

Interfaces that sense hand and arm gestures are widespread [4], including those that rely on motion sensors [5,6,7,8], changes in Bluetooth received signal strength [9], and light sensors [10,11]. Interfaces that leverage the movement of the legs and the feet have also been explored [12,13]. Computer vision-based approaches using the camera to capture head and body motions [14,15], facial expressions [16], and eye movement [17] also exist. 

Detection of throat activity has been explored using different enabling technologies. Acoustic sensors have been used for muscle movement recognition [18], speech recognition, [19] and actions related to eating [20,21,22]. Prior research has been done on e-textiles used in the neck region for detecting posture [23] and swallowing [24], but those efforts have relied on capacitive methods that have limitations in daily interactions. Researchers have explored sensoring the neck with piezoelectric sensors for monitoring eating [25] and medication adherence [26].

In addition to the neck-mounted sensors systems, there has been an exploration of actuation at the neck region using vibrotactile stimulation for accomplishing haptic perception [27,28,29]. 

The use of video image processing for speech recognition has been applied to lip reading [30,31,32]. More recently, as part of the silent or unvoiced speech recognition research efforts, mobile phone and wearable cameras have been used for speech classification from mouth movements. Researchers have used bespoke wearable hardware for detecting mouth and chin movements [33], or leveraged smart phone cameras [34].

Electromyography (EMG) has also been used for speech and/or silent speech classification. Researchers have used EMG sensors on the fingers placed on the face for mouth movement classifications [35]. EMG sensoring of the face for speech detection has also been carried out [36]. 

Tongue movement has been monitored for human–computer interfaces, including using a magnetometer to track a magnet in the mouth [37], using capacitive touch sensors mounted on a retainer in the mouth [38], using EMG from the face muscles around the mouth [39], and using EMG coupled with electroencephalography (EEG) as sensed from behind the ear [40]. Detecting tooth clicks has also been explored including a teeth-based interface that senses tooth clicks using microphones placed behind the ears [41].

Head position classification has been carried out with motion sensors on the head [42], pairing ultrasound transmitters and ultrasonic sensors mounted on the body [43] and barometric pressure sensing inside the ear [44]. 

This work is an expansion on our previously published conference paper [45] that classified head gestures using on a single neck-mounted bend sensor. In this expanded work, we look not only at head gesture classification using our neck-mounted sensor interface, but also at mouth movement classification, speech detection, and speech classification.

## 3. Prototype

**Figure 1:**
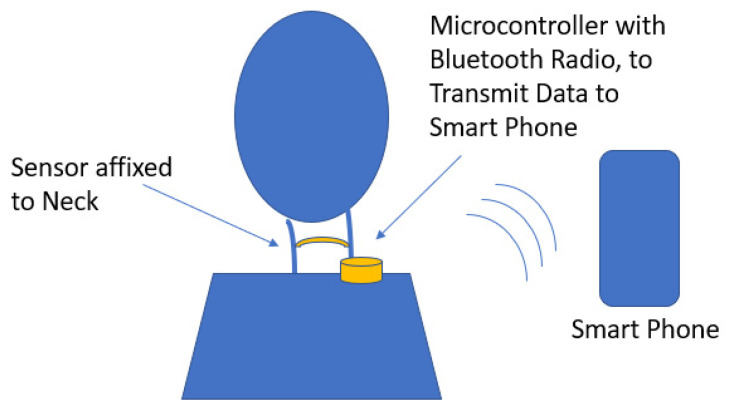
Prototype system’s component overview, with sensor placed on neck and wearable hardware placed on collar for communicating data to a smartphone for processing and for interfacing with the application.

A neck-mounted wearable prototype was developed and used for classifying neck movement, mouth movement, and speech. The prototype consists of a sensor affixed to the neck which is connected to a microcontroller. The data collected from the sensor is wirelessly transferred via Bluetooth by the microcontroller to the user’s paired smart phone. On the smart phone, the time-series data is in real time filtered, classified, and then used as input to a software application. Figure 1 provides an overview of the wearable system and its components interactions.

E-textile and flex sensors were investigated as potential candidates for the prototype. E-textiles can be used as capacitive sensors or as resistive sensors. With the capacitive method, the e-textile worked well as a proximity sensor to detect when the sensor was near human skin. However, once the sensor was in contact with or in close proximity of the skin, the sensor data became saturated and did not provide valuable features or respond to movements. Using the e-textile sensor as a resistive sensor was more successful in displaying features when actively bending or pulling the material.

The flex sensor proved to be the most appropriate for sensoring the neck. The flex sensor acts as a flexible potentiometer, whose resistance increases as the bend angle increases. Unlike the e-textile, which did not return to a static level after deformation and was prone to noise, the flex sensor performed reliably under bending and returned to a stable level when straight. 

A variety of positions for the sensor around the neck, chin, and side of face were explored with the neck being the most practical in terms of data collection and ease of wear. 

The hardware of the final prototype consists of an inexpensive (approximately USD 10) flex sensor, whose change in resistance signaled change in the bend of the sensor. The flex sensor was placed against the neck by weaving it under a small piece of paper that was taped to the neck. An Arduino microcontroller collected and wirelessly transmitted the data from the sensor to a smart phone for processing and display. Both an Arduino Nano and an Arduino Mega 2560 were used in the experiments. 

A simple moving average (SMA) filter was used to smooth the measured resistance signal. SMA filters replace the current data value with the unweighted mean of the k previous points in the data stream, in effect smoothing the data by flattening the impact of noise and artifact that is outside the bigger trend of the data. As the window size is decreased, the smoothness of the data is decreased. In this application, a window size that is too small can result in artifact and/or noise in the time-series data being improperly classified as a neck movement event. As the window size is increased, the impact of noise and artifact is also decreased, but the likelihood that relevant information is filtered out is increased. In this application, with a window size that is too large, there is the risk of delaying the recognition of neck movement events or even missing the events altogether. A window size of k = 40 was selected, which roughly maps to one second of data.

## 4. Head Tilt Detection

In a series of experiments, two types of flex sensors in a variety of positions on the neck are evaluated to determine the feasibility of differentiating and classifying head tilt and positioning.

In the experiments conducted, both a short sensor in three different positions and a long sensor were considered. Each sensor placement and sensor received 10 experiments per head-tilt with a time duration of 30 s. The tilts were held static for the entire 30 s. For each experiment, approximately 1100 data points were collected.

### 4.1. Flex Sensor Types and Placement

**Figure 2:**
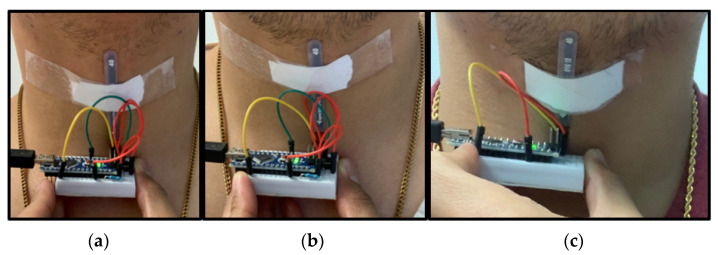
(**a**) Low, (**b**) center, and (**c**) high placement of the short flex sensor along the center line of the neck.

**Figure 3:**
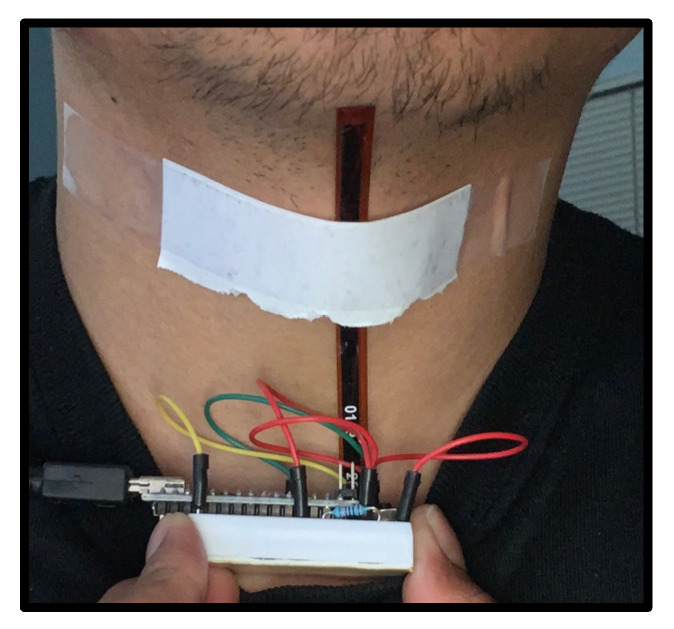
The placement of the long flex sensor along the center line of the neck.

Two types of flex sensors are considered: a short sensor and a long sensor. With the short sensor, three different placements are considered: a low placement, a center placement, and a high placement. The low placement is at the bottom of the neck, closest to the collar, as shown in Figure 2a. The center placement is directly over the larynx, at the middle of the neck, as shown in Figure 2b. The high placement is the top of the throat, closest to the chin, as shown in Figure 2c. The long sensor spans the three positions along the neck, from the base of the neck to under the chin, as shown in Figure 3.

### 4.2. Data Visualization

**Figure 4:**
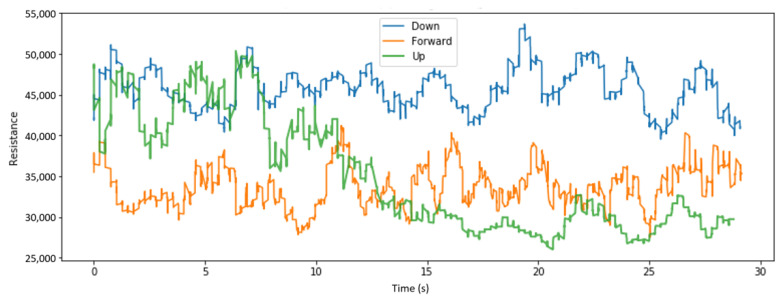
With low placement of short sensor, head tilt filtered data.

**Figure 5:**
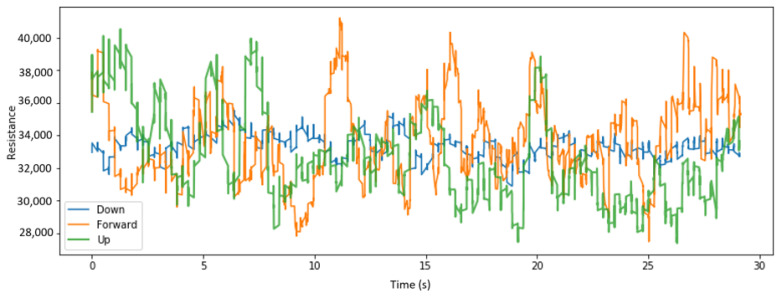
With center placement of short sensor, head tilt filtered data.

**Figure 6:**
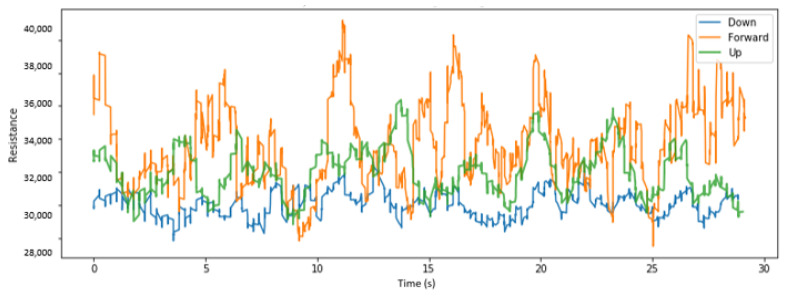
With high placement of short sensor, head tilt filtered data.

**Figure 7:**
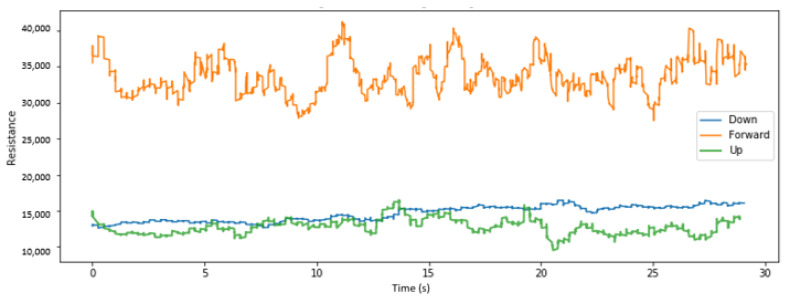
With long sensor, head tilt filtered data.

We visualize here some of the data collected across various placements of the sensors and for different head tilts. Figure 4, Figure 5 and Figure 6, respectively, display the collected resistance data over a 30-s time frame across the first three classes of head tilts, namely down, forward/no tilt, and up, for each placement of the short sensor, namely low, center, and high placement. Figure 7 displays the collected resistance data over a 30-s time frame for the long sensor, across the first three classes of head tilts, namely down, forward, and up. The data represented has been filtered using a moving average filter. 

**Figure 8:**
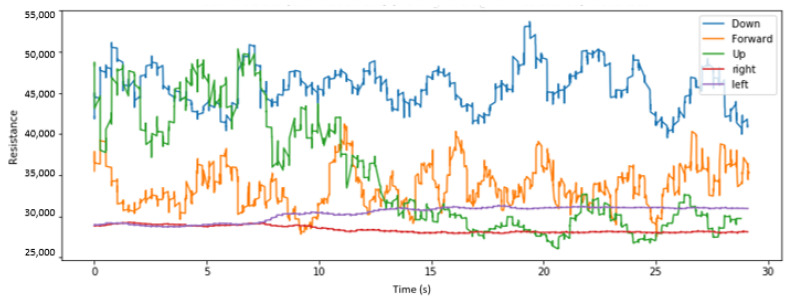
With low placement of short sensor, head tilt filtered data, with right and left tilts added.

**Figure 9:**
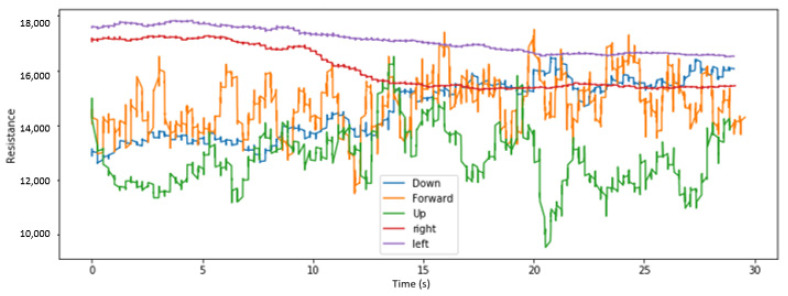
With long sensor, head tilt filtered data, with right and left tilts added.

The short, low sensor placement and the long sensor (Figure 4 and Figure 7, respectively) show the clearest distinction between the three classes. Therefore, the short, low sensor placement and the long sensor were further evaluated using all five classes of head tilts, namely down, forward, up, right, left. The collected resistance data over a 30-s time frame are shown in Figure 8 and Figure 9, respectively.

### 4.3. Head Tilt Detection Machine Learning Results

We evaluated the accuracy of classifying a three-class dictionary of head tilts. We then went on to evaluate the accuracy of classifying an expanded five-class dictionary of head tilts. The classification results are presented in this subsection. 

Three different classical machine learning (ML) classifiers were considered, specifically logistic regression, SVM, and random forest. The labeled dataset was partitioned into a train and held-out test set with an 80:20 ratio. To ensure the consistency of the models, a *k*-fold cross-validation was performed. A fivefold cross-validation of the train set was performed, with a random fourth of the examples in the training fold being used for validation during hyper-parameter tuning. For all the classical ML models, the Scikit-learn library in Python was used.

All four configurations, i.e., the long sensor and the three (low, center, and high) placements of the short sensor, were evaluated using the three head tilts (down, forward/not tilt, and up).

Table 1 displays our fivefold accuracy based on the model and placements of the sensors. In all cases, Logistic Regression was not sufficient in classifying the three-class dictionary. The short and low sensor placement and the long sensor had the best results. In both cases, random forest is the best performing model with test accuracies reaching ~83.4% and ~96% for the short, low placement and the long sensor, respectively.

To the best performing results, two additional classes were added. The two additional classes are the user’s head facing right and the user’s head facing left. 

Table 2 shows the performance of the short sensor with low placement and the long sensor when classifying against this five-class dictionary. As with previous results, random forest had the best performance with a test accuracy of ~83% for the short sensor and ~91% for the long sensor.

Table 3 shows the confusion matrix for the short sensor with low placement with the random forest classifier. The largest source of misclassifications are from the up data points, with only 65 out of 157 labels predicted correctly.

Table 4 shows the confusion matrix for the long sensor using the random forest classifier. With the long sensor, only 17 out of 182 up data points are mislabeled. The largest confusion is between left and right tilts.

From the confusion matrix the neck gesture language can be created. The most frequent or the most important gestures can be assigned to the head tilts that achieve the highest classification accuracy, both in terms of sensitivity and specificity. For example, the following mapping of neck gestures would be appropriate for the social media app Instagram. While on their feeds, users would tilt their heads forward to signal scrolling and would turn their heads to the side, either right or left, to ‘like’ an image.

## 5. Speech and Mouth Movement Detection

In this section, we explore a larger range of opportunities that the neck-mounted sensor can provide in addition to the head gesture detection detailed in Section 4. Section 5.1 addresses speech detection using the prototype, by differentiating speech from static breathing. Section 5.2 address mouth movement classification, namely the determination of how many times the mouth has been opened and closed. Section 5.3 tackles the challenging task of speech classification using only the detection of movement in the neck.

Speech and mouth movement detection provide contextual information that can be used to trigger or to mute the head tilt interface. For instance, if the system detects that the user is talking, then the user’s head tilts are not relayed to application software.

### 5.1. Speech Detection

**Figure 10:**
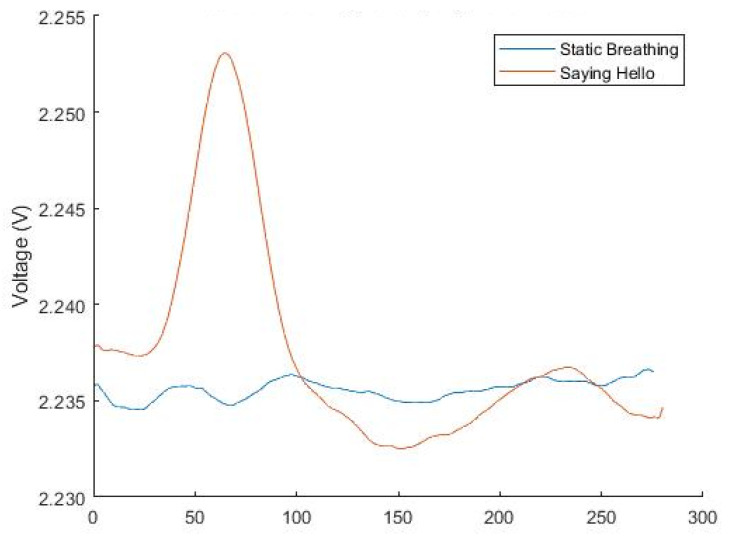
Sensor readings from static breathing and saying ‘hello’.

Figure 10 shows an example sensor reading from static breathing and from talking, specifically saying ‘hello’, on the same graph. The visualization demonstrates that the presence of speech can potentially be differentiated from static breathing using only the data collected from the flex sensor on the neck-mounted prototype. 

Using the neck-mounted prototype, an experiment was conducted to see if static breathing can indeed be differentiated from speech. Three-second-long samples with the prototype’s flex sensor were collected of both static breathing and of saying ‘hello’. A total of 60 samples, 30 of each class, were collected. The samples were classified using K-nearest neighbors (k-NN) with dynamic time warping (DTW), with k set to 3.

Dynamic time warping measures the similarity between two time-series signals, which may vary in speed and in length. It calculates the minimal distance between the signals allowing for warping of the time axis, with similar signals having lower cost than dissimilar signals.

Each test signal is compared against all the training signals, and the DTW cost between the test signal and each training signals is calculated. The DTW cost of the k nearest neighbors, i.e., most similar training signals, is then used to classify the signal.

Table 5 shows the confusion matrix for the classification results. The overall accuracy of the classification was 83.3% with 3 of the 30 talking samples misclassified as breathing.

### 5.2. Mouth Movement Classification

In another experiment, the classification of mouth movements without the generation of any sound was examined. The mouth was opened and closed without sound being generated. It was a four-class dictionary, with static breathing (no mouth movement), opening and closing of the mouth once, opening and closing of the mouth twice, and opening and closing of the mouth three times.

Three-second-long samples with the prototype’s flex sensor were collected with a total of 60 samples, 15 of each class. The samples were classified using K-nearest neighbors (k-NN) with dynamic time warping, with k set to 3. 

Table 6 shows the confusion matrix for the classification results. The overall accuracy of the classification was 67.5%. The classification of static breathing resulted in most of the misclassifications. By considering sample’s peak-to-valley amplitude, this misclassification can be decreased.

### 5.3. Speech Classification

The final experiments explored speech classification. Two different experiments of speech classification were carried with each having a set of four different sentences or phrases being spoken with the prototype affixed to the neck and the bend sensor capturing the neck activity.

For each of the two experiments, three-second-long samples with the prototype’s flex sensor were collected. For the first experiment with sentences, a total of 40 samples were collected, 10 of each class. The sentences used in the experiments were “I am a user who is talking right now”; “This is me talking with a sensor attached”; “Who am I talking to at this very moment?”; and “Can you recognize what I am saying while attached to a sensor?” For the second experiment with famous idioms, a total of 80 samples were collected, 20 of each class. The idioms used in the experiment were “a blessing in disguise”; “cut somebody some slack”; “better late than never”; and “a dime a dozen.” The samples were classified using K-nearest neighbors (k-NN) with dynamic time warping, with k set to 3. 

Table 7 and Table 8 show the confusion matrices for the classification results for the two experiments, respectively. The overall accuracy of the classification was 62.5% and 32.5%, respectively.

## 6. Discussion

The experiments with sensor data captured from the neck-mounted prototype show that the short sensor with low placement on the neck and the long sensor had the best results. For a three-class dictionary of head tilts, random forest is the best performing model with test accuracy of ~83.4% for the short sensor with low placement and ~96% for the long sensor. For a five-class dictionary of head tilts, random forest again had the best performance with a test accuracy of ~83% for the short sensor with low placement and ~91% for the long sensor.

Movements farther from the neck were also successfully detected and classified. Sensor data captured from the neck was able to differentiate speaking from static breathing, with ~83% accuracy. The presence and the number of mouth movements was classified with ~68% accuracy. Speech classification was more challenging, achieving up to 62.5% accuracy in differentiating spoken sentences from a four-class dictionary.

## 7. Conclusions

In this work, we show that subtle neck tilts, mouth movements, and speech can be detected and classified using an inexpensive flex sensor placed at the neck, and thus can prove to be enabling technology for use in software interfaces.

A flex sensor incorporated into a shirt collar or as part of a necklace opens new possibilities for software interaction. The accuracy of the classification of head tilts and their socially undisruptive nature makes head tilting a good option for signally software micro-interactions. For example, a tilt of the head can dismiss a smartwatch notification.

As head gestures can be made during the course of natural speech, the detection of speech and mouth movements allows for the interface to be tailored to times when a person is not speaking and thus improve the interface with greater context awareness.

## Figures and Tables

**Table 1:** Fivefold training, cross-validation, and held-out test accuracy of classical ML models with different feature sets. The bold font denotes the cases with the highest accuracy for that model. These results are for the three-class dictionary.

Model		Short Sensor Low Placement	Short Sensor Center Placement	Short Sensor High Placement	Long Sensor
Logistic Regression	*Train*	0.744	0.379	0.629	0.603
	*Validate*	0.74	0.379	0.622	0.602
	*Test*	0.76	0.349	0.589	0.608
SVM	*Train*	0.825	0.594	0.648	0.891
	*Validate*	0.809	0.547	0.612	0.881
	*Test*	0.824	0.555	0.575	0.891
Random Forest	*Train*	0.955	0.918	0.854	**0.989**
	*Validate*	0.821	0.665	0.694	**0.945**
	*Test*	0.834	0.669	0.671	**0.960**

**Table 2:** Fivefold training, cross-validation, and held-out test accuracy of classical ML models with different feature sets. The bold font denotes the cases with the highest accuracy for that model. These results are for the five-class dictionary that includes facing right and facing left.

Model		Short Sensor Low Placement	Long Sensor
Logistic Regression	*Train*	0.734	0.337
	*Validate*	0.733	0.338
	*Test*	0.755	0.363
SVM	*Train*	0.756	0.869
	*Validate*	0.741	0.812
	*Test*	0.76	0.818
Random Forest	*Train*	0.956	**0.977**
	*Validate*	0.824	**0.915**
	*Test*	0.828	**0.91**

**Table 3:** Five-class confusion matrix for the short sensor with low placement. Rows represent actual class and columns represent predicted class.

Random Forest		Predicated				
		Down	Forward	Up	Right	Left
Actual	Down	259	0	10	0	0
	Forward	1	285	40	1	3
	Up	25	47	65	15	5
	Right	0	0	8	185	18
	Left	0	0	3	29	194

**Table 4:** Five-class confusion matrix for the long sensor. Rows represent actual class and columns represent predicted class.

Random Forest		Predicated				
		Down	Forward	Up	Right	Left
Actual	Down	202	3	11	0	0
	Forward	0	494	2	0	0
	Up	17	0	182	0	0
	Right	0	0	0	204	49
	Left	0	0	0	36	166

**Table 5:** Two-class confusion matrix for static breathing and talking. Rows represent actual class and columns represent predicted class.

		Predicated	
		Static Breathing	Talking
Actual	Static Breathing	23	7
	Talking	3	27

**Table 6:** Four-class confusion matrix for mouth movements. Rows represent actual class and columns represent predicted class.

		Predicated			
		Breathing	One Cycle	Two Cycles	Three Cycles
Actual	Breathing	2	3	3	12
	One cycle	0	19	1	0
	Two cycles	0	7	13	0
	Three cycles	0	0	0	20

**Table 7:** Four-class confusion matrix for spoken sentences. Rows represent actual class and columns represent predicted class.

		Predicated			
		“I Am …”	“This Is …”	“Who …”	“Can You …”
Actual	“I am a user who is talking right now.”	0	9	1	0
	“This is me talking with a sensor attached.”	0	10	0	0
	“Who am I talking to at this very moment?”	0	4	6	0
	“Can you recognize what I am saying while attached to a sensor?”	0	0	1	9

**Table 8:** Four-class confusion matrix for spoken phrases. Rows represent actual class and columns represent predicted class.

		Predicated			
		“A Blessing in Disguise”	“Cut Somebody Some Slack”	“Better Late than Never”	“A Dime a Dozen”
Actual	“A blessing in disguise”	0	0	14	6
	“Cut somebody some slack”	0	2	1	17
	“Better late than never”	0	0	19	1
	“A dime a dozen”	0	0	15	5
